# Pharmacological and Non-Pharmacological Postoperative Pain Management Practices Among Nurses in Vietnam: A Cross-Sectional Study

**DOI:** 10.3390/nursrep16040106

**Published:** 2026-03-25

**Authors:** Van Hoi Le, Huu Thuan Vo, Thi Bich Thuy Tran, My Hanh Dang, Cai Thi Thuy Nguyen, Thi Anh Nguyen

**Affiliations:** 1 School of Medicine, Tan Tao University, Tay Ninh 80000, Vietnam; hoi.le@ttu.edu.vn (V.H.L.); thuy.tran@ttu.edu.vn (T.B.T.T.); 2 Cho Ray Hospital, Ho Chi Minh City 70000, Vietnam; thuanvh@ntt.edu.vn; 3 Faculty of Nursing, Nguyen Tat Thanh University, Ho Chi Minh City 70000, Vietnam; 4 Faculty of Nursing—Midwifery, Hong Bang International University, Ho Chi Minh City 70000, Vietnam; hanhdm@hiu.vn; 5 Research Institute for the Humanities and Social Sciences, National Science and Technology Council, Taiwan/Center for the Advancement of the Humanities and Social Sciences, National Taiwan University, Taipei 10617, Taiwan; nguyenthuycai@hmu.edu.vn; 6 Faculty of Nursing and Midwifery, Hanoi Medical University, Hanoi 100000, Vietnam

**Keywords:** postoperative pain management, nursing practice, knowledge–attitude–practice, non-pharmacological interventions, Vietnam

## Abstract

**Background/Objectives:** Despite extensive research on nurses’ knowledge and attitudes toward pain management globally, limited evidence exists regarding the actual implementation of multimodal pain management practices among Vietnamese nurses. This study aimed to (1) assess nurses’ implementation of pharmacological and non-pharmacological postoperative pain management interventions, (2) examine the relationships among knowledge, attitude, and practice (KAP), and (3) identify predictors of competent practice with attention to the relative contributions of formal training versus clinical experience. **Methods:** A cross-sectional survey was conducted among 230 nurses working in Urology Departments from two tertiary public hospitals in Ho Chi Minh City, Vietnam, between April and June 2024, focusing on postoperative pain management. Pain management knowledge, attitudes, and practices were assessed using validated instruments. Independent samples *t*-tests compared trained versus untrained nurses. Multiple linear regression identified predictors of practice competency. Effect sizes (Cohen’s d) quantified the magnitude of training effects. **Results:** Nurses demonstrated moderate-to-good competency, with pharmacological interventions (M = 3.74) implemented more consistently than non-pharmacological interventions (M = 3.48, *p* < 0.001). Trained nurses significantly outperformed untrained nurses across all domains with large effect sizes (Cohen’s d = 1.34–1.54). A clear hierarchy emerged in non-pharmacological practice: environmental (M = 4.01) > physical (M = 3.69) > cognitive–behavioral (M = 3.27) > spiritual (M = 2.60). Strong KAP correlations were observed (r = 0.70–0.85, *p* < 0.001). Prior training was the strongest predictor of both pharmacological (β = 1.31, *p* < 0.001) and non-pharmacological practice (β = 0.58, *p* < 0.001), while clinical experience showed no significant effect (*p* > 0.40). **Conclusions:** This study provides evidence that formal training—not clinical experience—is strongly associated with competent postoperative pain management practice among Vietnamese nurses, with large effect sizes demonstrating practical significance. The strong KAP relationships support targeted educational interventions addressing knowledge gaps to improve practice. These findings have implications for nursing education research in Vietnam and similar healthcare settings.

## 1. Introduction

Pain remains a major global health concern requiring continuous attention from healthcare professionals. Effective pain management significantly impacts patients’ quality of life, functional capacity, and psychological well-being [1,2]. Nurses play a vital role in pain assessment and management, serving as primary caregivers who coordinate both pharmacological and non-pharmacological interventions [3].

Contemporary evidence supports multimodal pain management, which integrates pharmacological approaches (analgesics, opioids, adjuvant medications) with non-pharmacological strategies (physical comfort measures, cognitive–behavioral techniques, relaxation, and spiritual support). This comprehensive approach is essential because reliance on any single modality often proves insufficient [4,5]. However, studies have reported persistent gaps in nurses’ pain management practices [6,7]. While 83.3% of nurses reported regular pain assessment in one study, only 32.2% used standardized tools such as the numeric rating scale [8], and 77.8% lacked positive attitudes toward pain management [9]. Non-pharmacological interventions, despite their evidence base and safety profile, remain underutilized globally [5,10].

The Knowledge–Attitude–Practice (KAP) framework provides a theoretical foundation for understanding factors influencing healthcare behaviors [11]. According to this framework, knowledge acquisition leads to attitude formation, which subsequently influences practice. While previous studies in Vietnam have examined nurses’ knowledge and attitudes toward pain management separately [8,9], there is a paucity of research examining: (1) the actual practice of both pharmacological and non-pharmacological interventions, (2) the interrelationships among KAP domains, and (3) predictors of competent practice. Furthermore, no study has quantified the magnitude of training effects using standardized effect sizes, limiting the ability to assess practical significance beyond statistical significance.

This study addresses these gaps by comprehensively examining postoperative pain management practices among nurses caring for post-surgical patients in urology departments. Specifically, this study aimed to: (1) assess nurses’ implementation of both pharmacological and non-pharmacological postoperative pain management interventions; (2) examine the relationships among knowledge, attitude, and practice (KAP); (3) compare practice competency between nurses with and without formal pain management training; and (4) identify predictors of competent practice with attention to the relative contributions of formal training versus clinical experience. Based on the existing literature and KAP framework, we hypothesized that: (H1) Trained nurses will demonstrate significantly higher scores in both pharmacological and non-pharmacological practice domains compared to untrained nurses; (H2) Strong positive correlations will exist among knowledge, attitude, and practice scores; and (H3) Prior formal training will be a stronger predictor of practice competency than clinical experience alone.

## 2. Materials and Methods

### 2.1. Study Design and Setting

This cross-sectional study was conducted at two tertiary public hospitals in Ho Chi Minh City, Vietnam: Cho Ray Hospital (an 1800-bed national referral center) and a second major public hospital. Both institutions serve as regional referral centers with high surgical volumes and standardized nursing protocols. The study setting was selected to capture pain management practices in high-acuity environments representative of tertiary care in Vietnam.

Study Endpoints and Experimental Protocol: The primary endpoints of this study were: (1) mean pharmacological and non-pharmacological pain management practice scores among nurses; (2) difference in practice scores between trained and untrained nurses; and (3) significant predictors of practice. No experimental intervention was administered; this was a purely observational cross-sectional survey. Data were collected via a self-administered structured questionnaire at a single time point (April–June 2024), following ethics approval and hospital administration consent.

### 2.2. Participants

Eligible participants were registered nurses working in the Urology Departments of the study hospitals who met the following inclusion criteria: (1) current employment as a registered nurse, (2) minimum one year of experience providing direct care to post-surgical patients, and (3) willingness to participate. Nurses on leave during the data collection period or those with administrative-only roles were excluded.

Exclusion criteria were: (1) nurses who declined to provide informed consent; (2) nurses who were on extended leave during the data collection period (April–June 2024); and (3) incomplete questionnaire responses (defined as >20% missing items). A total of 241 nurses were approached; 11 were excluded (5 declined consent, 6 had incomplete responses), yielding a final analytical sample of 230 participants.

Training status was determined by self-reported completion of formal pain management education programs. Nurses classified as “trained” had completed at least one structured educational program on pain management, including continuing professional development (CPD) courses, hospital-based training workshops, or university-level pain management modules. These programs typically covered pain assessment tools, pharmacological protocols, non-pharmacological techniques, and ethical considerations in pain care, with duration ranging from one-day workshops to semester-long courses. Training content was relevant to the urology surgical context, including postoperative pain assessment and multimodal analgesia. Nurses classified as “untrained” had not completed any formal pain management education beyond their basic nursing curriculum.

### 2.3. Study Instruments

The study utilized a self-administered questionnaire consisting of four parts:

**Part I: Demographic characteristics** included age, sex, highest educational attainment (diploma, bachelor’s, master’s degree), years of clinical experience, and prior pain management education.

**Part II: Knowledge assessment** consisted of 29 items covering pain physiology, assessment, and management principles. Each correct response scored 1 point (range: 0–29).

**Part III: Attitude assessment** included 14 items measuring personal beliefs about pain, patient assessment attitudes, and management philosophies, rated on a 5-point Likert scale.

**Part IV: Practice assessment** was adapted from Menlah et al. (2018) [12], consisting of 15 items: non-pharmacological interventions (10 items) covering environmental comfort, physical modalities, cognitive–behavioral techniques, and spiritual support; and pharmacological interventions (5 items) covering medication administration practices. Each item was rated on a 5-point Likert scale (1 = never to 5 = always).

The adapted Vietnamese version underwent rigorous validation: (1) forward translation by two bilingual nursing experts, (2) back-translation by an independent bilingual expert, (3) content validity review by a panel of five nursing experts (Content Validity Index, CVI = 0.89), and (4) pilot testing with 30 nurses (not included in the main study). The instrument demonstrated strong internal consistency reliability (Cronbach’s alpha = 0.87 for pharmacological practice; alpha = 0.91 for non-pharmacological practice subscales). These psychometric properties confirm the questionnaire’s validity and reliability for use in this Vietnamese clinical nursing population.

### 2.4. Data Collection Procedure

Data were collected between April and June 2024. After obtaining hospital approval, eligible nurses were identified from departmental staff rosters and randomly selected. Selected nurses were individually approached during shift changes, provided with study information sheets, and given time to consider participation. Those who agreed signed informed consent forms before completing the questionnaire. Questionnaire completion took approximately 15–20 min.

### 2.5. Ethical Considerations

The study received ethical approval from the Human Research Ethics Committee of Cho Ray Hospital (approval No. 1747/CN-HDDD). Participants were fully informed about the study’s purpose, voluntary nature, and their right to withdraw. Written informed consent was obtained from all participants. Anonymity was maintained throughout data collection and analysis.

### 2.6. Data Analysis

Sample size was calculated using G*Power software (version 3.1.9.2). With an expected effect size of 0.20, power of 90%, and 5% margin of error for multiple regression with six predictors, the required sample was 209 participants. To account for potential 10% rate of invalid or incomplete responses, 230 nurses were recruited using simple random sampling from staff rosters. All 230 recruited nurses completed the survey (response rate: 100%).

Statistical analyses were performed using Jamovi software (version 2.3.21) for descriptive statistics, independent samples *t*-tests, one-way ANOVA, and Pearson correlation analyses, and Python (version 3.11) with statsmodels, scipy, and matplotlib libraries for multiple linear regression modeling, effect size calculations, and figure generation. Categorical variables were summarized as frequencies and percentages. Continuous variables were presented as mean ± standard deviation (SD).

**Group comparisons:** Independent samples *t*-tests compared practice scores between trained and untrained nurses. Cohen’s d effect sizes were calculated to quantify the magnitude of differences, interpreted as small (0.2), medium (0.5), or large (0.8) effects [13]. One-way ANOVA compared practice scores across education levels with post hoc Tukey tests.

**Correlation analysis:** Pearson correlation coefficients examined relationships among knowledge, attitude, and practice domains.

**Regression analysis:** Two multiple linear regression models were conducted—one for pharmacological practice and one for non-pharmacological practice—with demographic variables and prior training as predictors. Assumptions were verified including linearity, independence (Durbin–Watson), homoscedasticity, multicollinearity (VIF < 10), and normality of residuals.

Statistical significance was set at *p* < 0.05 (two-tailed).

## 3. Results

### 3.1. Participant Characteristics

Table 1 presents the demographic characteristics of the 230 participating nurses. The mean age was 37.3 years (SD = 6.67, range 22–53), with an average of 13.7 years of clinical experience (SD = 5.84). The majority were female (81.3%), which is consistent with the Vietnamese nursing workforce where women constitute approximately 80–85% of registered nurses nationally, reflecting historical and cultural patterns in the nursing profession. Bachelor’s degree holders comprised the largest group (84.8%). Notably, 57.4% had not received any formal pain management training beyond their basic nursing curriculum.

### 3.2. Pain Management Practices: Overall Findings

Overall, nurses demonstrated moderate-to-good pain management competency. Pharmacological interventions (M = 3.74, SD = 0.49) were implemented more consistently than non-pharmacological interventions (M = 3.48, SD = 0.50). This difference was statistically significant (paired t = 8.86, *p* < 0.001), indicating systematic prioritization of medication-based approaches.

### 3.3. Impact of Prior Training on Practice

**Figure 1:**
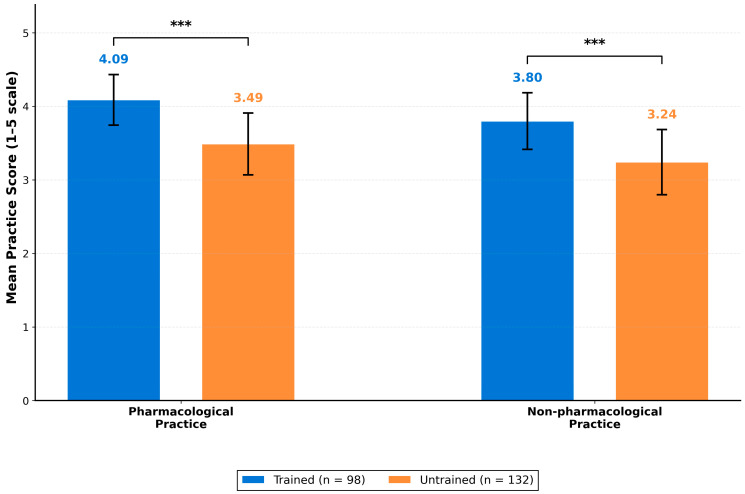
Comparison of pain management practice scores between trained and untrained nurses. Error bars represent standard deviation. *** *p* < 0.001.

To examine the effect of formal training, we compared mean scores between trained (*n* = 98) and untrained (*n* = 132) nurses (Table 2, Figure 1). Trained nurses demonstrated significantly higher scores across all practice domains with large effect sizes. For pharmacological practice, trained nurses scored substantially higher (M = 4.09, SD = 0.37) than untrained nurses (M = 3.49, SD = 0.42), with this difference being statistically significant (t = 11.55, *p* < 0.001) and representing a large effect size (Cohen’s d = 1.54). Similarly, non-pharmacological practice scores were significantly higher among trained nurses (M = 3.80, SD = 0.41) compared to untrained nurses (M = 3.24, SD = 0.43; t = 10.01, *p* < 0.001, Cohen’s d = 1.34). The training effect extended beyond practice behaviors to underlying competencies: knowledge scores differed markedly between trained (M = 22.11, SD = 2.89) and untrained nurses (M = 17.11, SD = 2.91; t = 12.95, *p* < 0.001, Cohen’s d = 1.73), as did attitude scores (trained: M = 3.85, SD = 0.35; untrained: M = 3.33, SD = 0.38; t = 10.66, *p* < 0.001, Cohen’s d = 1.42). All effect sizes exceeded Cohen’s threshold for large effects (d > 0.80), indicating that formal training has substantial practical significance. Trained nurses scored approximately 1.0–1.7 standard deviations higher than untrained colleagues.

### 3.4. Hierarchy of Non-Pharmacological Interventions

**Figure 2:**
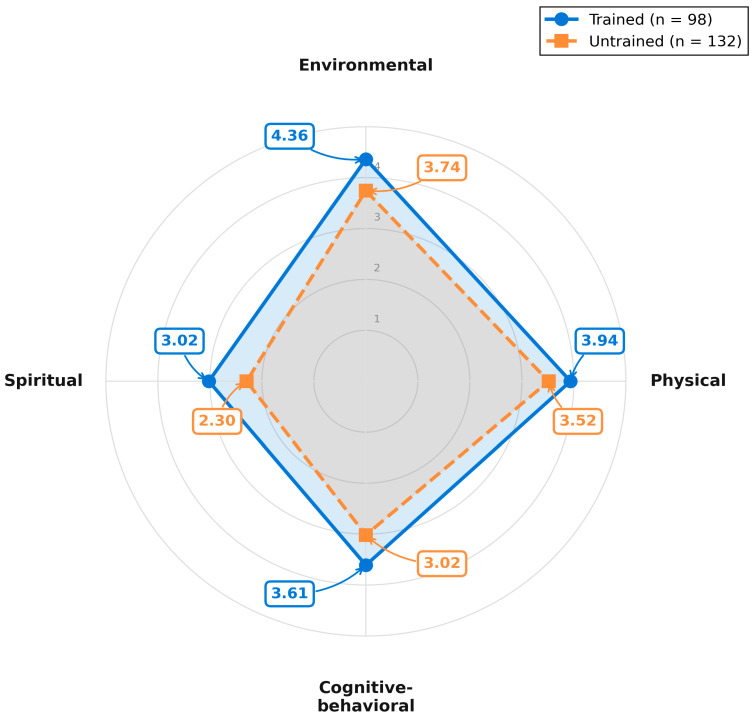
Radar chart showing non-pharmacological practice profiles by training status across four intervention categories.

**Figure 3:**
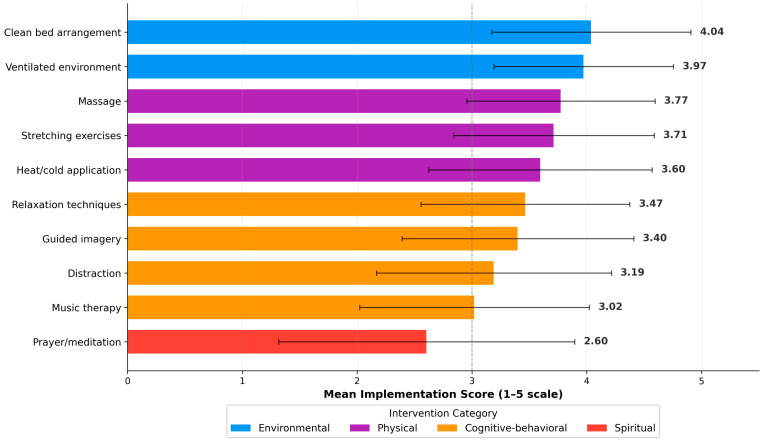
Hierarchy of non-pharmacological pain management interventions ranked by mean implementation score. Dashed line indicates moderate level (score = 3). Color coding: blue = environmental, purple = physical, orange = cognitive–behavioral, red = spiritual.

Analysis of non-pharmacological practice revealed a clear four-tier hierarchy (Figure 2 and Figure 3). Environmental comfort interventions ranked highest, including clean bed arrangement (M = 4.04, SD = 0.87) and ventilated environment (M = 3.97, SD = 0.78), followed by physical modalities such as massage (M = 3.77, SD = 0.82), stretching (M = 3.71, SD = 0.87), and heat/cold application (M = 3.60, SD = 0.97). Cognitive–behavioral techniques showed moderate-to-low implementation: relaxation (M = 3.47, SD = 0.91), guided imagery (M = 3.40, SD = 1.01), distraction (M = 3.19, SD = 1.02), and music therapy (M = 3.02, SD = 1.00). Spiritual support through prayer/meditation encouragement was least implemented (M = 2.60, SD = 1.29). Trained nurses outperformed untrained nurses across all categories, with the largest effects for cognitive–behavioral techniques (d = 1.08) and environmental comfort (d = 0.97).

### 3.5. Pharmacological Pain Management Practices

Table 3 presents pharmacological practices. Scheduled opioid administration (M = 3.90, SD = 0.75), dose titration (M = 3.85, SD = 0.74), and PRN medication (M = 3.84, SD = 0.78) were consistently implemented. An ethically concerning finding emerged: placebo injection use to verify pain authenticity (M = 3.34, SD = 1.07) showed high variability, indicating substantial disagreement among nurses.

### 3.6. Knowledge–Attitude–Practice (KAP) Relationships

**Figure 4:**
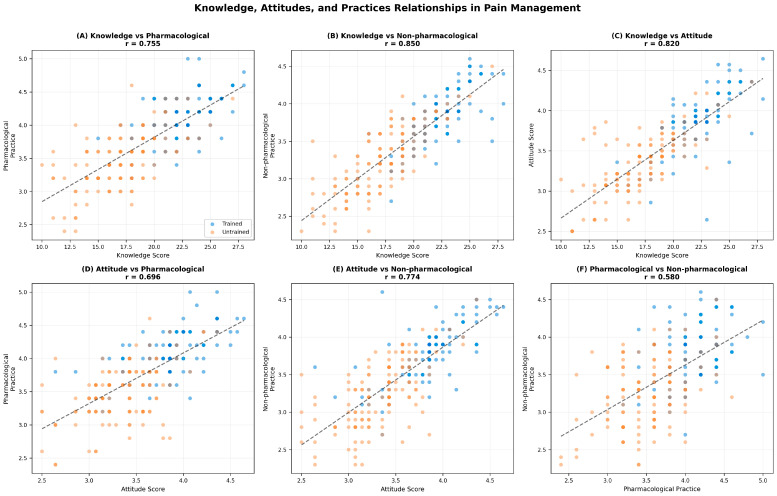
Knowledge-Attitude-Practice (KAP) relationships. (**A**) Knowledge vs. pharmacological practice; (**B**) Knowledge vs. non-pharmacological practice; (**C**) Knowledge vs. attitude; (**D**) Attitude vs. pharmacological practice; (**E**) Attitude vs. non-pharmacological practice; (**F**) Pharmacological vs. non-pharmacological practice. Blue dots = trained nurses; red dots = untrained nurses. Solid lines represent linear regression fits. All correlations significant at *p* < 0.001.

Pearson correlation analysis revealed strong positive associations among KAP domains (Figure 4). Knowledge was strongly correlated with both pharmacological (r = 0.76, *p* < 0.001) and non-pharmacological practice (r = 0.85, *p* < 0.001), while attitude showed similar patterns with pharmacological (r = 0.70, *p* < 0.001) and non-pharmacological practice (r = 0.77, *p* < 0.001). Knowledge and attitude were also highly correlated (r = 0.82, *p* < 0.001). These strong correlations support the KAP theoretical framework. Notably, knowledge was more strongly associated with non-pharmacological practice than pharmacological practice, suggesting non-pharmacological interventions are more knowledge-dependent.

### 3.7. Predictors of Pain Management Practice

**Figure 5:**
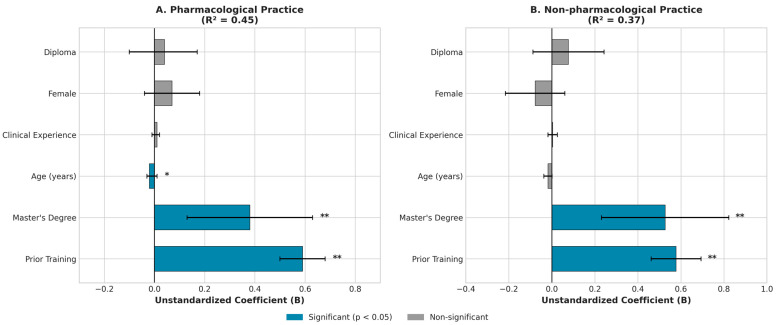
Forest plot showing predictors of pain management practice. (**A**) Pharmacological practice; (**B**) Non-pharmacological practice. Blue bars indicate significant predictors (*p* < 0.05); gray bars indicate non-significant predictors. * *p* < 0.05; ** *p* < 0.01. Error bars represent 95% confidence intervals.

Multiple linear regression identified predictors of both practice domains (Table 4, Figure 5). For pharmacological practice (R^2^ = 0.45, F = 30.12, *p* < 0.001), prior training was the strongest predictor (B = 0.59, β = 1.31, *p* < 0.001), followed by master’s education (B = 0.38, *p* = 0.003), while age showed a small negative association (B = −0.02, *p* = 0.033) and clinical experience was not significant (*p* = 0.442). Non-pharmacological practice (R^2^ = 0.37, F = 22.08, *p* < 0.001) showed similar patterns: prior training (B = 0.58, *p* < 0.001) and master’s education (B = 0.53, *p* = 0.001) were significant predictors, while clinical experience again showed no effect (*p* = 0.718). Across both domains, formal training and advanced education—not clinical experience—predicted competent practice.

## 4. Discussion 

### 4.1. Study Contributions

This study provides important contributions to the understanding of postoperative pain management in Vietnam, building on and extending existing KAP literature in the regional and global context. First, it offers a comprehensive assessment of both pharmacological and non-pharmacological pain management practices among Vietnamese nurses, revealing that prior formal training—not clinical experience—was strongly associated with competent practice across all domains. Second, it documents the hierarchy of non-pharmacological intervention implementation and identifies specific practice gaps, consistent with international evidence [14,15]. Third, the large effect sizes (Cohen’s d = 1.34–1.54) provide practical significance beyond statistical significance, suggesting meaningful differences between trained and untrained nurses in clinical practice.

### 4.2. The Critical Role of Formal Training

The most notable finding is that prior formal training was strongly associated with both pharmacological and non-pharmacological practice competency, while years of clinical experience showed no significant association. However, this cross-sectional association should be interpreted cautiously: nurses who self-select into training programs may differ systematically from those who do not in motivation, institutional support, or baseline competency. Unmeasured institutional factors—such as ward leadership, staffing levels, and continuing education culture—may also confound this relationship. The effect sizes were large (Cohen’s d = 1.34–1.54), suggesting substantial practical differences, but prospective studies with pre-post designs are needed to establish whether training itself produces these differences.

This finding challenges the assumption that nursing competencies naturally develop through on-the-job experience. Instead, it suggests that experiential learning alone may be insufficient for developing evidence-based pain management skills, and that structured educational interventions may be essential [16,17]. However, longitudinal studies are needed to confirm whether formal training programs produce sustained improvements in clinical competency.

### 4.3. KAP Relationships and Educational Implications

The strong KAP correlations support the theoretical framework that knowledge and attitude are prerequisites for practice. Notably, knowledge was more strongly correlated with non-pharmacological practice (r = 0.85) than pharmacological practice (r = 0.76). This suggests that non-pharmacological interventions—which require understanding of psychological principles and therapeutic techniques—are more knowledge-dependent than medication administration.

These findings have direct implications for curriculum design: educational programs should emphasize cognitive–behavioral techniques and spiritual care approaches, which showed the largest implementation gaps despite being evidence-based interventions [5,10].

### 4.4. Hierarchy of Non-Pharmacological Interventions

The documented hierarchy (environmental > physical > cognitive > spiritual) aligns with international findings [10,18,19] and reflects practical constraints. Environmental and physical comfort measures require minimal training and resources. In contrast, cognitive–behavioral techniques require specialized knowledge and time, while spiritual care may involve cultural sensitivity and personal discomfort.

The finding that training was most strongly associated with improved cognitive–behavioral practice (d = 1.08) suggests that educational interventions specifically targeting these underutilized evidence-based techniques could potentially improve postoperative pain care quality. It is important to note, however, that the role and relevance of non-pharmacological interventions may differ substantially between postoperative and chronic pain settings. In chronic pain management, cognitive–behavioral and psychological approaches are often central components of interdisciplinary care [20], whereas in the acute postoperative context studied here, their implementation may be more limited by practical constraints such as time, patient readiness, and clinical priorities.

### 4.5. Ethical Concerns

The reported use of placebo injections to verify pain authenticity (M = 3.34, SD = 1.07) raises substantial ethical concerns that warrant careful consideration. This practice violates several fundamental principles of clinical ethics, including respect for patient autonomy, beneficence, and the obligation not to deceive patients [7]. The International Association for the Study of Pain (IASP) defines pain as a subjective experience and explicitly discourages the use of placebos to “test” pain authenticity [21]. Similarly, the American Pain Society guidelines emphasize that pain assessment should rely on patient self-report as the most reliable indicator [22]. The use of deceptive placebos undermines the therapeutic relationship, erodes patient trust, and may contribute to the undertreatment of pain [23]. However, it is important to acknowledge that respondents may have interpreted this questionnaire item differently than intended or may have reported practices that reflect institutional norms rather than personal clinical decisions. The high variability in responses (SD = 1.07) suggests substantial disagreement among nurses regarding this practice. Furthermore, cultural and institutional contexts in Vietnam may influence the prevalence of such practices. Regardless of interpretation, these findings highlight an urgent need for explicit ethics education within pain management curricula, addressing deception in clinical care and reinforcing patient-centered assessment principles consistent with international nursing codes of ethics [24].

### 4.6. Limitations

Several limitations should be acknowledged and carefully considered when interpreting these findings. First, the cross-sectional design precludes causal inferences; the observed associations between training and practice competency cannot establish that training produces improved practice. Nurses who pursue training may differ from those who do not in ways not captured by our measures (self-selection bias). Second, practice data were entirely based on self-report questionnaires, which introduces critical methodological limitations. Social desirability bias is a major concern—nurses may systematically overreport adherence to evidence-based practices and underreport ethically problematic behaviors (e.g., placebo use). Recall bias may further compromise the accuracy of practice frequency estimates. Crucially, self-reported practice scores should not be equated with actual clinical behavior; the KAP-to-practice gap in real clinical settings may be substantially larger than documented here. These limitations are particularly significant for interpreting statistically elevated practice scores, as observed means may reflect perceived or aspirational rather than actual practice patterns. Future research must employ direct behavioral observation, patient medical record audits, or patient-reported pain outcomes to obtain objective practice measures. Third, the sample was restricted to urology departments in two tertiary public hospitals in one metropolitan area (Ho Chi Minh City), limiting generalizability to other clinical specialties, chronic pain settings, rural or community hospitals, private institutions, or non-tertiary facilities. Fourth, the study did not assess patient outcomes (e.g., pain scores, satisfaction, length of stay), so the clinical impact of the practice differences observed cannot be determined. Fifth, this study focused specifically on postoperative pain management in surgical urology patients; findings may not extend to chronic pain management or other acute pain contexts. Sixth, the classification of nurses as “trained” or “untrained” was based on self-report and encompassed heterogeneous training experiences of varying duration, format, and content quality, which may obscure important differences within these groups.

Second, while the KAP framework guided this study’s conceptual design, its limitations must be acknowledged. The KAP model has been criticized for oversimplification—it assumes that knowledge acquisition linearly translates to attitude change and then to practice improvement [11]. As discussed in medical anthropology literature, knowledge alone does not automatically translate into behavior change. Important contextual determinants not captured in this study include: institutional protocols, physician prescribing patterns, staffing levels and workload constraints, hospital culture, national opioid regulations, and available resources. Future studies should employ more comprehensive theoretical frameworks (e.g., the Theory of Planned Behavior or the COM-B model) to capture these contextual barriers to practice implementation.

## 5. Conclusions

This cross-sectional study provides evidence that formal pain management training—not accumulated clinical experience—is strongly associated with competent postoperative pain management practice among Vietnamese nurses, with large effect sizes demonstrating practical significance. The strong knowledge–attitude–practice correlations support the theoretical importance of educational interventions that address knowledge gaps to improve clinical practice. These findings suggest that structured educational programs warrant investigation through prospective interventional studies to determine whether targeted training can causally improve pain management outcomes. Future implementation research should examine whether multimodal educational interventions—particularly those emphasizing cognitive–behavioral techniques and ethical pain assessment—can improve patient-centered pain care in similar tertiary hospital settings in low- and middle-income countries. The ethical concerns regarding placebo use underscore the need for explicit ethics education within pain management curricula.

## Figures and Tables

**Table 1:** Participant characteristics (N = 230).

Characteristic	*n* (%) or Mean (SD, Range)
**Age (years)**	37.3 (6.67, 22–53)
**Work experience (years)**	13.7 (5.84, 1–28)
**Sex**	
Male	43 (18.7)
Female	187 (81.3)
**Educational level**	
Diploma	27 (11.7)
Bachelor’s	195 (84.8)
Master’s	8 (3.5)
**Prior pain management training**	
Yes	98 (42.6)
No	132 (57.4)

SD = Standard Deviation.

**Table 2:** Comparison of knowledge, attitude, and practice between trained and untrained nurses (N = 230).

Variable	Trained (*n* = 98)	Untrained (*n* = 132)	t	*p*	Cohen’s d
Pharmacological Practice	4.09 (0.37)	3.49 (0.42)	11.55	**<0.001**	**1.54**
Non-pharmacological Practice	3.80 (0.41)	3.24 (0.43)	10.01	**<0.001**	**1.34**
*Environmental*	4.36 (0.60)	3.74 (0.67)	7.25	**<0.001**	0.97
*Physical*	3.94 (0.54)	3.52 (0.63)	5.28	**<0.001**	0.70
*Cognitive–behavioral*	3.61 (0.51)	3.02 (0.56)	8.06	**<0.001**	**1.08**
*Spiritual*	3.02 (1.20)	2.30 (1.27)	4.38	**<0.001**	0.58
Knowledge Score	22.11 (2.89)	17.11 (2.91)	12.95	**<0.001**	**1.73**
Attitude Score	3.85 (0.35)	3.33 (0.38)	10.66	**<0.001**	**1.42**

Values are Mean (SD). Independent samples *t*-test; df = 228 for all comparisons. Cohen’s d effect size interpretation: 0.2 = small, 0.5 = medium, 0.8 = large. All comparisons significant at *p* < 0.001. Bold values indicate large effects (d ≥ 0.80). Italics indicate ethically concerning practice.

**Table 3:** Pharmacological pain management practices (N = 230).

Practice Item	Mean (SD)
I give opioids on a regular schedule	3.90 (0.75)
I administer subsequent doses to suit individual patient’s response	3.85 (0.74)
I give analgesics when the patient asks for medication	3.84 (0.78)
I encourage early ambulation/exercise with analgesia	3.80 (0.71)
*I give sterile water by injection (placebo) to determine if pain is real*	3.34 (1.07)

SD = Standard Deviation. Items rated 1 (never) to 5 (always). Italics indicate ethically concerning practice.

**Table 4:** Multiple linear regression: Predictors of pain management practice (N = 230).

Predictor	Pharmacological (R^2^ = 0.45)				Non-Pharmacological (R^2^ = 0.37)			
	B	SE	β	*p*	B	SE	β	*p*
Prior training	0.59	0.05	1.31	**<0.001**	0.58	0.06	1.16	**<0.001**
Master’s degree	0.38	0.13	0.85	**0.003**	0.53	0.15	1.05	**0.001**
Age	−0.02	0.01	−0.26	**0.033**	−0.02	0.01	−0.24	0.064
Clinical experience	0.01	0.01	0.09	0.442	0.004	0.01	0.05	0.718

B = unstandardized coefficient; SE = standard error; β = standardized coefficient. Bold *p*-values indicate *p* < 0.05.

## Data Availability

Data available on request from the corresponding author. The data are not publicly available due to privacy and ethical restrictions.

## Public Involvement Statement

Patients and the public were not involved in the design, conduct, reporting, or dissemination of this research. This study involved only registered nursing staff as participants in their professional capacity.

## Guidelines and Standards Statement

This cross-sectional study was conducted and reported in accordance with the STROBE (Strengthening the Reporting of Observational Studies in Epidemiology) guidelines for cross-sectional studies.
